# Identifying reproducible individual differences in childhood functional brain networks: An ABCD study

**DOI:** 10.1016/j.dcn.2019.100706

**Published:** 2019-09-19

**Authors:** Scott Marek, Brenden Tervo-Clemmens, Ashley N. Nielsen, Muriah D. Wheelock, Ryland L. Miller, Timothy O. Laumann, Eric Earl, William W. Foran, Michaela Cordova, Olivia Doyle, Anders Perrone, Oscar Miranda-Dominguez, Eric Feczko, Darrick Sturgeon, Alice Graham, Robert Hermosillo, Kathy Snider, Anthony Galassi, Bonnie J. Nagel, Sarah W. Feldstein Ewing, Adam T. Eggebrecht, Hugh Garavan, Anders M. Dale, Deanna J. Greene, Deanna M. Barch, Damien A. Fair, Beatriz Luna, Nico U.F. Dosenbach

**Affiliations:** a Department of Psychiatry, Washington University in St. Louis, St. Louis, MO, 63110, USA; b Department of Psychology, University of Pittsburgh, Pittsburgh, PA 15213, USA; c Department of Medical Social Sciences, Northwestern University, Chicago, IL 60611, USA; d Department of Neurology, Washington University in St. Louis, St. Louis, MO, 63110, USA; e Department of Psychiatry, University of Pittsburgh, Pittsburgh PA, 15213, USA; f Departments of Psychiatry & Behavioral Neuroscience, Oregon Health and Science University, Portland, OR 97213, USA; g Department of Medical Informatics and Clinical Epidemiology, Oregon Health and Science University, Portland, OR, 97213, USA; h Mallinckrodt Institute of Radiology, Washington University in St. Louis, St. Louis, MO, 63110, USA; i Department of Psychiatry, University of Vermont, Burlington VT 05401, USA; j Department of Radiology, University of California San Diego, San Diego, CA, USA; k Department of Psychological and Brain Sciences, Washington University in St. Louis, St. Louis, MO, USA; l Department of Biomedical Engineering, Washington University in St. Louis, St. Louis, MO, 63110, USA; m Department of Pediatrics, Washington University in St. Louis, St. Louis, MO, 63110, USA; n Department of Radiology, Washington University in St. Louis, St. Louis, MO, 63110, USA

**Keywords:** ABCD, Resting state fMRI, Functional connectivity, Development, Cognitive ability, Reproducibility

## Abstract

The 21-site Adolescent Brain Cognitive Development (ABCD) study provides an unparalleled opportunity to characterize functional brain development via resting-state functional connectivity (RSFC) and to quantify relationships between RSFC and behavior. This multi-site data set includes potentially confounding sources of variance, such as differences between data collection sites and/or scanner manufacturers, in addition to those inherent to RSFC (e.g., head motion). The ABCD project provides a framework for characterizing and reproducing RSFC and RSFC-behavior associations, while quantifying the extent to which sources of variability bias RSFC estimates. We quantified RSFC and functional network architecture in 2,188 9-10-year old children from the ABCD study, segregated into demographically-matched discovery (N = 1,166) and replication datasets (N = 1,022). We found RSFC and network architecture to be highly reproducible across children. We did not observe strong effects of site; however, scanner manufacturer effects were large, reproducible, and followed a “short-to-long” association with distance between regions. Accounting for potential confounding variables, we replicated that RSFC between several higher-order networks was related to general cognition. In sum, we provide a framework for how to characterize RSFC-behavior relationships in a rigorous and reproducible manner using the ABCD dataset and other large multi-site projects.

## 1 Introduction

Adolescence is a unique developmental period of specialization during which neural processes supporting goal-directed behavior continue to stabilize towards adult levels (Larsen and Luna, 2018). Importantly, adolescents are particularly vulnerable to the onset of major psychiatric illnesses such as schizophrenia, mood disorders, and addiction (Paus et al., 2008). Predominant neurodevelopmental models (Di Martino et al., 2014; Grayson and Fair, 2017; Luna et al., 2015) hypothesize that maturation of widely-distributed patterns of resting-state functional connectivity (RSFC) within and between functional networks underlie individual differences in cognitive development and that RSFC disruption is associated with the emergence of psychiatric disorders during adolescence. Work on person-specific patterns of RSFC suggests that the functional connectome uniquely reflects an individual's trait level features (Gratton et al., 2018) and thus may be predictive of individual differences in cognitive function and psychopathology. However, due to challenges in recruiting neuroimaging samples representative of the full range of behavioral variability in the population, a direct and comprehensive mapping between individual variability in functional networks and trait level features has remained elusive. The ABCD dataset (Volkow et al., 2018), which has recruited close to 12,000 participants for brain imaging and behavioral assessments, affords a critical opportunity for a more systematic mapping of the associations between RSFC and behavior.

Associations between functional brain networks and behavior often employ RSFC, which is a particularly useful approach for delineating the functional architecture of infra-slow blood-oxygen-level-dependent (BOLD) activity. Both group- and individual-level RSFC studies in adults have revealed high reproducibility of functional networks subserving primary sensorimotor functions, as well as stable and adaptive forms of control and attention (Gordon et al., 2017, 2016; Laumann et al., 2015; Power et al., 2011; Yeo et al., 2011). From the perspective of lifespan development, components of association networks, including the default mode, attention, and control networks can be observed in utero (Thomason et al., 2013; van den Heuvel and Thomason, 2016), though their organization continues to mature throughout the first few years of life (Gao et al., 2015; Smyser et al., 2010; Wheelock et al., 2019). While the fundamental aspects of functional organization are established prior to adolescence (Grayson et al., 2014; Marek et al., 2015; Power et al., 2012), this is a unique period for neural modification, as distributed patterns of RSFC, particularly cross-network integration, display a protracted maturation throughout childhood and adolescence and into adulthood (Grayson et al., 2014; Marek et al., 2015; Nielsen et al., 2018; Power et al., 2012).

Supporting the potential behavioral relevance of developmental patterns in functional networks, between network connectivity of the “task-negative” default mode network and “task-positive” dorsal attention network has been shown to correlate with individual differences in general cognitive ability (Anticevic et al., 2012; Shine et al., 2019; Smith et al., 2015). Nevertheless, we still lack a comprehensive understanding of the links between RSFC and developmentally-sensitive behavioral phenomena, including the stabilization of goal-directed behavior. The identification of RSFC-behavior associations is particularly challenging in pediatric populations due to greater behavioral variability compared to adults (Montez et al., 2017). Children also exhibit greater levels of head motion, which have been shown to be associated with RSFC and several cognitive and demographic variables (Siegel et al., 2017). This limited understanding of brain-behavior relationships prevents the translation of RSFC studies to potential therapeutic targets for intervention.

The need for increased reproducibility in RSFC and the inconsistency of brain-behavior relationships within the existing literature is likely driven, in part, by the reliance on small sample sizes, which have low statistical power (Button et al., 2013). Small sample sizes are also limited in their ability to fully represent the relevant variability of individual differences in behavior across populations. To this end, neuroimaging samples, like those used in behavioral research, tend to be biased towards Caucasian, western, educated, industrialized, rich, and democratic groups (Henrich et al., 2010). Moreover, inconsistency in the reported literature is also likely driven by descriptive analytic approaches that draw inferences from a single sample, rather than an analytic focus on result reproducibility and improved generalization (Poldrack et al., 2017).

The Adolescent Brain Cognitive Development (ABCD) study has the ability to overcome the shortcomings of small sample sizes, enabling the generation of more reproducible and generalizable results in a sample of nearly 12,000 children (Volkow et al., 2018). Yet, acquiring large quantities of human MRI data is non-trivial, requiring the harmonization of scanner sequences across three different manufacturers (Siemens, GE, and Philips) and 21 data collection sites (Casey et al., 2018). The extent to which differences in scanners affect estimates of brain function and relationships with key variables (e.g., age) remains debated (Auzias et al., 2016; Focke et al., 2011; Noble et al., 2017). Therefore, the current project aimed to quantify and contrast RSFC between manufacturers, data collection site, and other biologically relevant variables such as sex.

Leveraging data collected from the large 21-site developmental ABCD study, the current project provides a framework of analytic approaches to quantify results reproducibility (hereafter referred to as reproducibility for brevity; see (Goodman et al., 2016)) in RSFC, which is critical for studies examining neurodevelopmental RSFC-behavioral associations. First, we provide an overview of the functional network architecture in the 9–10 year old child brain and address potential confounding variables, including scanner manufacturer and data collection site. We also explore sex differences, as boys and girls have been suggested to follow divergent pathways of brain maturation (Bellis and De Bellis, 2001). Next, we provide an example approach for relating RSFC with cognitive data, utilizing analytical approaches aimed at increasing reproducibility. Specifically, we build on the broad brain-behavior literature by examining the relationship between RSFC and neuropsychological performance (general cognitive function) in discovery and replication datasets, with over 1,000 participants in each dataset. For each analysis, we conducted statistical testing at the network level using enrichment analysis (Eggebrecht et al., 2017) to correct for multiple comparisons across networks, which is an analytical technique originally developed for genome wide association studies (Backes et al., 2014). All analyses were performed on demographically matched split-halves of the sample, in order to examine reproducibility. Taken together, the current work characterizes RSFC and network architecture in the child brain, quantifies key potential confounding variables inherent to large-scaled multi-site studies, and provides a framework for producing rigorous and reproducible brain-behavior associations.

## 2 Material and methods

### 2.1 Sample

This project utilized a dataset consisting of RSFC data from N = 3,694 participants through the ABCD fast track portal and behavioral data from 11,572 participants from the ABCD 2.0 release (Volkow et al., 2018). To obtain the final sample size, children from the full behavioral sample (N = 11,572) were first divided into a discovery (N = 5,786) and replication (N = 5,786) sets (Table 1), which were matched across 10 variables: site location, age, sex, ethnicity, grade, highest level of parental education, handedness, combined family income, and exposure to anesthesia. Family members (e.g., sibling pairs, twins, and triplets) were kept together in the same set and the two sets were matched to include equal numbers of single participant and family members (Table 1).

**Table 1:** Demographic Information.

	Discovery (N = 1,166)	Replication (N = 1,022)
Age (months)	*M =* 120.49, *SD* = 7.35	*M* = 120.5, *SD* = 7.59
Sex	635 Female | 531 Male	509 Female | 513 Male
Sibling Status: N(%) (unrelated/sibling/twin/triplet)	773/150/240/1 (66.3/12.9/20.8/0.1%)	652/146/220/4 (63.8%/14.3%/21.5%/0.4%)
Framewise Displacement (mm; across all uncensored frames)	*M =* 0.11, *SD* = 0.04, range = (0.03-0.199)	*M* = 0.11, *SD* = 0.04, range = (0.03-0.199)
Average Frames Included	*M* = 1,294, *SD* = 187	*M* = 1,309, *SD* = 180
NIH Toolbox Total	*M* = 88.52, *SD* = 8.23 (N = 1142)	*M* = 88.64, *SD* = 8.53 (N = 998)

Head motion can systematically bias developmental studies (Power et al., 2012; Siegel et al., 2017), as well as those relating RSFC to behavior (Siegel et al., 2017). However, these systematic biases can be addressed through rigorous head motion correction (Power et al., 2014). Therefore, we used strict inclusion criteria with regard to head motion in the current study. Specifically, inclusion criteria for the current project (see (Casey et al., 2018) for broader ABCD inclusion criteria) consisted of an average frame-wise displacement (FD; described below) across all neuroimaging runs of < 0.20 and at least 600 frames (8 min) of low-motion (FD < 0.20) RSFC data. Based on these criteria, our final dataset consisted of RSFC data from a total of N = 2,188 youth across the discovery (N = 1,166) and replication (N = 1,022) sets. The final discovery and replication sets did not differ in mean FD (Δ*M* = 0.01, *t* = 1.17, *p* =  0.24), total frames included (Δ*M* = 14, *t* = 1.81, *p* =  0.07), or NIH Toolbox total (Δ*M* = 0.10, *t* = 0.27, *p* =  0.79). There was a significant difference between sex ($\chi^{2}$ = 4.73, *p* = 0.03); however, we noted negligible-to-small effects of sex on RSFC and included it as a covariate in models assessing the relationship between RSFC and behavior. We note that 24 participants were missing data for the NIH toolbox cognitive measures in the discovery and replication set. For RSFC-behavior relationships using the NIH toolbox (see Material and Methods Section 2.10), only participants with complete behavioral data were included in these analyses, resulting in a sample of 1,142 in the discovery set and 998 in the replication set (Table 1).

### 2.2 MRI acquisition

Imaging for each youth was performed across 21 sites within the United States, harmonized across Siemens Prisma, Philips, and GE 3 T scanners. Details on image acquisition can be found in (Casey et al., 2018). Twenty minutes of eyes-open (passive crosshair viewing) resting state data were presented to ensure at least 8 min of low-motion data. All resting state scans were acquired using a gradient-echo EPI sequence (TR =800 ms, TE =30 ms, flip angle = 90°, voxel size = 2.4 mm^3^, 60 slices). Head motion was monitored online using Framewise Integrated Real-time MRI Monitor (FIRMM) software at Siemens sites (Dosenbach et al., 2017).

### 2.3 HCP-style CIFTI processing overview

All processing was completed with the newly released and freely available ABCD-HCP pipelines (https://github.com/DCAN-Labs/abcd-hcp-pipelines). The ABCD-HCP pipelines are modified from the original HCP pipelines (Glasser et al., 2013). Briefly, this pipeline comprises six stages. 1) PreFreesurfer normalizes anatomical data. This normalization entails brain extraction, denoising, and then bias field correction on anatomical T1 and/or T2 weighted data. The ABCD-HCP pipeline includes two additional modifications to improve output image quality. ANTs DenoiseImage models scanner noise as a Rician distribution and attempts to remove such noise from the T1 and T2 anatomical images. Additionally, ANTs N4BiasFieldCorrection attempts to smooth relative image histograms in different parts of the brain and improves bias field correction. 2) FreeSurfer constructs cortical surfaces from the normalized anatomical data. This stage performs anatomical segmentation, white/grey and grey/CSF cortical surface construction, and surface registration to a standard surface template. Surfaces are refined using the T2 weighted anatomical data. Midthickness surfaces, which represent the average of white/grey and grey/CSF surfaces, are generated here. 3) PostFreesurfer converts prior outputs into an HCP-compatible format (i.e. CIFTIS) and transforms the volumes to a standard volume template space using ANTs nonlinear registration, and the surfaces to the standard surface space via spherical registration. 4) The “Vol” stage corrects for functional distortions via reverse-phase encoding spin-echo images. All resting state runs underwent intensity normalization to a whole brain mode value of 1,000, within run correction for head movement, and functional data registration to the standard template. Atlas transformation was computed by registering the mean intensity image from each BOLD session to the high resolution T1 image, and then applying the anatomical registration to the BOLD image. This atlas transformation, mean field distortion correction, and resampling to 3-mm isotropic atlas space were combined into a single interpolation using FSL’s applywarp tool (Smith et al., 2004). 5) The “Surf” stage projects the normalized functional data onto the template surfaces which is described below. 6) We have added an fMRI and fcMRI preprocessing stage, “DCANBOLDproc” which is also described below.

### 2.4 fMRI “Surf” processing

The BOLD fMRI volumetric data are sampled to each participant’s original mid-thickness left and right-hemisphere surfaces constrained by the grey-matter ribbon as described in (Glasser et al., 2013). Once sampled to the surface, timecourses were deformed and resampled from the individual’s original surface to the 32k fs_LR surface in a single step. This resampling allows point-to-point comparison between each individual registered to this surface space. These surfaces were then combined with volumetric subcortical and cerebellar data into the CIFTI format using Connectome Workbench (Marcus et al., 2011), creating full brain timecourses excluding non-gray matter tissue. Finally, the resting-state timecourses were smoothed with 2 mm full-width-half-maximum (FWHM) kernel applied to geodesic distances on surface data and euclidean distances on volumetric data.

### 2.5 “DCANBOLDproc” preprocessing

Additional preprocessing steps to reduce spurious variance unlikely to reflect neuronal activity were executed as recommended in (Ciric et al., 2017; Power et al., 2014). First, a respiratory filter is used to improve FD estimates calculated in the “vol” stage. Second, temporal masks were created to flag motion-contaminated frames using the improved FD estimates (Power et al., 2012). Frames with FD > 0.20 mm were flagged as motion-contaminated. After computing the temporal masks for high motion frame censoring, the data were processed with the following steps: (i) demeaning and detrending, (ii) interpolation across censored frames using least squares spectral estimation of the values at censored frames (Power et al., 2014) so that continuous data can be (iii) denoised via a GLM including: whole brain, ventricular, and white matter signals, as well as their derivatives. Denoised data are then passed through (iv) a band-pass filter (0.008 Hz < f < 0.10 Hz) without re-introducing nuisance signals (Hallquist et al., 2013) or contaminating frames near high motion frames (Carp et al., 2013).

### 2.6 Generation of vertex-wise functional networks

The network organization of the group-average cortical surface in both datasets was derived using the graph-theory-based Infomap algorithm for community detection (Rosvall and Bergstrom, 2008), following (Power et al., 2011). In this approach, we calculated the Pearson correlation matrix of the timecourses from all cortical vertices (n = 59,412), averaged across participants. Correlations between vertices within 30 mm of each other were set to zero. Geodesic distance was used for within-hemisphere surface connections. Inter-hemispheric connections between the cortical surfaces were retained, as smoothing was not performed across the mid-sagittal plane.

This matrix was then thresholded at a range of values calculated based on the resulting density of the matrix as in (Gordon et al., 2017; Marek et al., 2018); the density thresholds ranged from .1% to 5%. Small networks with 400 or fewer vertices were considered unassigned and removed from further consideration as in (Gordon et al., 2017). Putative network identities were then assigned to each participant’s communities similar to previously published work from our group (Gordon et al., 2017). Reproducibility between discovery and replication datasets was quantified using normalized mutual information (NMI). Values range from 0 to 1, with 0 indicating no overlap in network structure and 1 indicating complete overlap of network structure between datasets. Additionally, we quantified the degree (% difference) to which the current ABCD network assignments differed from a previously defined adult Infomap-generated network assignment.

### 2.7 Generation of parcellated functional networks

We extracted the time series of RSFC data from a recent parcellation of 333 ROIs covering the entire cortical surface (Gordon et al., 2016). This parcellation was chosen because it comprises major cortical functional networks, including control networks, processing networks, and the default mode network and covers the entire cortical surface. This parcellation has been shown to exceed many others with respect to homogeneity of the BOLD signal within each parcel (Gordon et al., 2016; Miranda-Dominguez et al., 2018). All subsequent analyses were run on parcel-level RSFC data.

### 2.8 Analyses of scanner, site, and sex related effects

The ABCD dataset is uniquely suited to reveal population level variability in relationships between brain and behavior; however, the multi-vendor and multi-site nature of ABCD lends it vulnerable to biases related to these factors. We employed several approaches to quantify RSFC differences as a function of scanner manufacturer (SIemens, Philips, and GE), including similarity analyses to quantify the degree of shared RSFC variance within and between scanner manufacturers, multidimensional scaling, one-way analysis of variance (ANOVA) to contrast regional effects of scanner manufacturer, and pearson correlation to determine the relationship between edgewise RSFC/scanner manufacturer correlations (assessed by point biserial correlation) and euclidean distance between ROI pairs.

To quantify shared RSFC variance across each individual, we correlated each edge of each participant’s correlation matrix with every other participant, resulting in a participant x participant similarity matrix (Gratton et al., 2018). To address the associations of RSFC with potential covariates and other demographic variables, we separately sorted these matrices by acquisition site, scanner type, and sex, and quantified the effect size in differences in shared variance of each parameter.

We used multidimensional scaling (MDS) approaches to depict how scanner, site, and sex variance affected RSFC in a data driven fashion. MDS places data in multidimensional space based on the similarity, as measured by Euclidean distance among data points (correlation-based distances produce similar results). RSFC matrices from each participant were entered into an MDS algorithm (implemented using Matlab 2018b, *cmdscale.m*) and grouped by scanner, site, and sex, separately. Subsequently, we averaged these distances across subjects to visualize the mean estimate for the examined variable and computed its standard error in two dimensional space.

To quantify the effect of scanner manufacturer at each edge in RSFC matrices across subjects, we conducted a one-way ANOVA of scanner manufacturer for each ROI pair. To visualize the average effect of scanner manufacturer (Fig. 2C), for each ROI, we averaged the F-statistics from that ROI to every other ROI. After noting negligible to small differences between Philips and GE scanners (Fig. 2A and B), we collapsed subjects across these manufacturers and subsequently employed point biserial correlations at each edge to quantify the relationship between scanner manufacturer and RSFC. We then regressed these resulting correlations against Euclidean distance between ROIs to test for systematic distance-dependent biases.

We observed a significant difference (p < 0.05) in average FD between subjects scanned in Siemens (Mean FD = 0.10) vs. Philips (Mean FD = 0.13) scanners and between Philips and GE (Mean FD = 0.10) scanners, but not between Siemens and GE scanners (*p* = 0.97), indicating there may be differences in the scanner noise floor for Philips scanners. Future studies should probe this area further. We also suggest future studies may consider tailoring FD cut-offs on a per scanner manufacturer basis.

We contrasted 32- vs. 64-channel head coil across Siemens scanners in the discovery and replication dataset, separately. All sites using GE and Philips scanners used 32-channel head coils. Of the 13 Siemens sites, 4 sites used a scanner equipped with a 64-channel head coil (discovery: 64-channel coil N = 119, 32-channel coil N = 665; replication: 64-channel coil N = 101, 32-channel coil N = 601). We conducted a similarity analysis (as in Fig. 2A) and subsequently determined the effect size (Cohen’s D) for the difference in similarity between subjects scanned using a 32-channel vs. 64-channel head coil.

Recent studies suggest other potential confounding factors, such as time of year, may affect RSFC (Di et al., 2019). Here, we gathered site altitude to test for potential confounding effects of altitude on RSFC/scanner associations as a function of anatomical distance for each pairwise scanner manufacturer comparison. Specifically, we gathered altitude data for each of the 21 ABCD sites. We conducted two analyses: (1) we included site altitude as a covariate in comparisons of distance-dependent effects between scanner manufacturers (pairwise); and (2) removed sites (N = 2) that were of substantially higher altitude than all other sites and subsequently repeated the analysis correlating RSFC manufacturer correlations with anatomical distance between each manufacturer pair.

### 2.9 Between-participant RSFC variability

Between-participant variance in RSFC was assessed across the cortex as the standard deviation of Fischer z-transformed correlations between a given region and every other region across participants (Laumann et al., 2015). To subsequently quantify this variability for each region, we averaged the between-participant variability across all correlations involving a given region. We assessed whether between-participant variability differs across scanner manufacturers by calculating the standard deviation of correlations across participants imaged using each scanner manufacturer and subsequently calculating the effect size (Cohen’s d) of the difference in standard deviations between each scanner pair. In addition to variability within ROI pairs and networks, we also examined connectome-wide patterns of between-participant variability by performing principal component analysis (PCA, MATLAB’s singular value decomposition algorithm) on a matrix composed of all ROI x ROI pairs from each participant. Over two hundred components (243 in discovery, 221 in replication) had eigenvalues that exceeded those from a parallel analysis of resampled data (5,000 iterations). In the current project, we present the first 10 components from this analysis, which combined to explain over 10% (11.88% in discovery, 11.73% in replication) of the variance among the 59,412 ROI x ROI pairs across patiricpants (Fig. S1) and were similar in number to traditionally reported functional networks in large group-average and single-subject analyses (i.e., between 7–17 functional networks (Yeo et al., 2011; Power et al., 2011; Laumann et al., 2015; Gordon et al., 2016, 2017).

### 2.10 Brain-behavior correlations

We tested for correlations between RSFC and cognition by using total “Cognition Composite” scores from the NIH toolbox cognition battery (see (Luciana et al., 2018) for overview of ABCD cognitive assessments). The use of these “total scores” allowed us to assess RSFC association with a highly reliable measure of individual differences in cognition (test-retest *r* = .90, see (Heaton et al., 2014)). This measure is inclusive of multiple domains of cognitive function (Weintraub et al., 2013), hereafter referred to as general cognitive ability. Only participants with complete behavioral data were included in these analyses, resulting in samples of 1,142 in the discovery set and 998 in the replication set. We note that within the final analysis sample, which included rigorous head motion thresholding, there was a weak, but significant relationships between FD and general cognitive ability in the discovery set (*r* = −0.07, *p* =  0.02) that was not reproduced in the replication set (*r* = −0.04, *p* =  0.21).

In order to first examine the overall pattern of associations between RSFC and general cognitive ability, we regressed the NIH toolbox total scores onto RSFC for each ROI pair, while covarying scanner manufacturer, which had a more appreciable effect than data collection site, and sex, each coded as categorical varialbes, within a multiple linear regression model (fitlm in Matlab). As above, in order to examine reproducibility of any observed effects, these models were run separately on the discovery and replication sets. This resulted in an ROI x ROI matrix of brain-behavior relationships, represented by the t-statistic associated with the toolbox total predictor from the multiple regression. Here, split-half reliability of brain behavior relationships was examined by correlating the t-statistics across all edges between the discovery and replication sets. We note the estimate of split-half reliability was relatively unchanged when performing this analysis on coefficients rather than t-statistics.

To examine the extent to which specific within- or between-network connections were associated with general cognitive ability, enrichment analysis was used to quantify significance of network-level effects. These analyses paralleled those published previously in RSFC-behavior analyses (Eggebrecht et al., 2017; Marrus et al., 2018; McKinnon et al., 2019; Wheelock et al., 2019) and were originally adopted from genome-wide association studies (Backes et al., 2014; Khatri et al., 2012; Rivals et al., 2007). First, correlations between Fisher-z-transformed RSFC and total scores were calculated separately across participants for each ROI-pair (N = 55,278) within the discovery and replication set. We then applied an uncorrected p-threshold of 0.05 to the resulting RSFC-cognition correlations resulting in a binarized, nominally thresholded RSFC-cognition association matrix. Next, we used a hypergeometric test to assess enrichment within- and between-network pairs of suprathreshold associations between RSFC and cognition. The hypergeometric test assesses network-pair enrichment by comparing observed associations against the total number of associations observed across the entire matrix, in addition to the total possible associations within a given network pair. Connectome-wide permutation-based p-values were estimated by randomly permuting the subject labels corresponding to NIH toolbox total scores, correlating this with RSFC, and calculating false hypergeometric values for each network pair 10,000 times and then pooling the null values across the full set of network pairs. Significance of network-pairs was determined using false discovery rate correction (Backes et al., 2014; Eggebrecht et al., 2017).

## 3 Results

### 3.1 Network architecture and between-participant variability

**Fig. 1:**
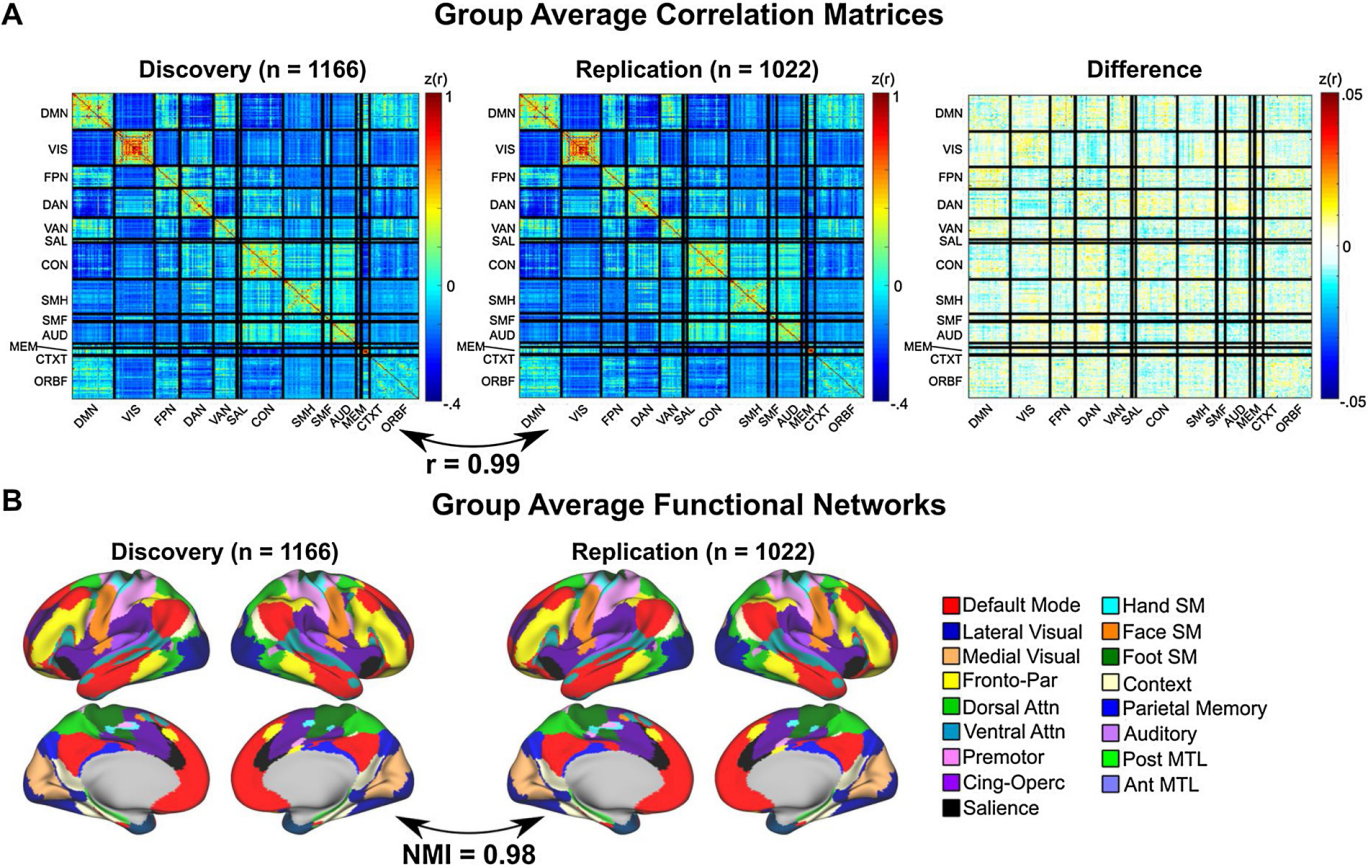
**ABCD RSFC and functional network architecture is highly reproducible. (A)** Group average correlation matrices in a discovery and replication set, and the difference (Discovery - Replication). Color bar represents Fisher Z-transformed correlations between ROIs. **(B)** Group average functional networks. The correlation across every ROI pair between the discovery and replication dataset was *r* = 0.99. Functional network architecture was similarly highly reproducible with a normalized mutual information value (NMI) of 0.98. Fronto-Par = Frontoparietal; Dorsal Attn = Dorsal Attention; Ventral Attn = Ventral Attention; Cing-Oper = Cingulo-opercular; Hand SM = Hand Somatomotor; Face SM = Face Somatomotor; Foot SM = Foot Somatomotor; Post MTL = Posterior medial temporal lobe; Ant MTL = Anterior medial temporal lobe.

We used a previously defined functional parcellation of 333 ROIs (Gordon et al., 2016) that span the cortical surface in a total sample of 2,188 individuals, aged 9–10 years within the ABCD sample. These 2,188 individuals were separated into a discovery data set (N = 1,166) and a replication data set (N = 1,022), matched across several demographics (Table 1). For each participant, we correlated the time series of each ROI with that of every other ROI creating connectivity matrices for each participant, and subsequently formed group average matrices by averaging across participants within each data set. Group average correlation matrices for the discovery and replication datasets are present in Fig. 1A. These matrices were nearly identical (*r* = 0.99), with only very small differences (*r* ˜ 0.01) in correlation for any given edge. Thus, in the ABCD dataset, highly reproducible group-averaged RSFC is observed at these sample sizes.

The sorting of ROIs into previously described adult functional network architecture (Gordon et al., 2016) resulted in the appearance of the canonical block structure of RSFC observed in adults. To delineate the functional network architecture within the ABCD dataset, we employed the Infomap community detection algorithm (see Material and Methods 2.6) to group-averaged vertex-wise data in the discovery and replication set (Fig. 1B). To quantify reproducibility, we calculated normalized mutual information (NMI) between the resulting output vectors, finding nearly full reproducibility in network structure between the discovery and replication datasets (NMI = 0.98).

We quantified the degree of overlap between the group-averaged ABCD vertex-wise network solution with a previously-defined group-average adult network solution (Gordon et al., 2017). Fig. S2A depicts where there is a difference between ABCD and Gordon et al Infomap assignments (black surface vertices in Fig. S2A). In total, 29% of all vertices demonstrated a different network assignment between ABCD and Gordon et al datasets. Many of these were along network boundaries, which may be a result of small differences in alignment from processing streams (Fig. S2B). When we masked out network borders, only 13% of the remaining vertices demonstrated differing network assignments between ABCD and Gordon et al., indicating adult network topology is largely present by late childhood (Marek et al., 2015). That said, because these solutions are not exact, this network solution is publically available for other ABCD researchers (https://dosenbachlab.wustl.edu/data).

### 3.2 Quantification of potential covariates

**Fig. 2:**
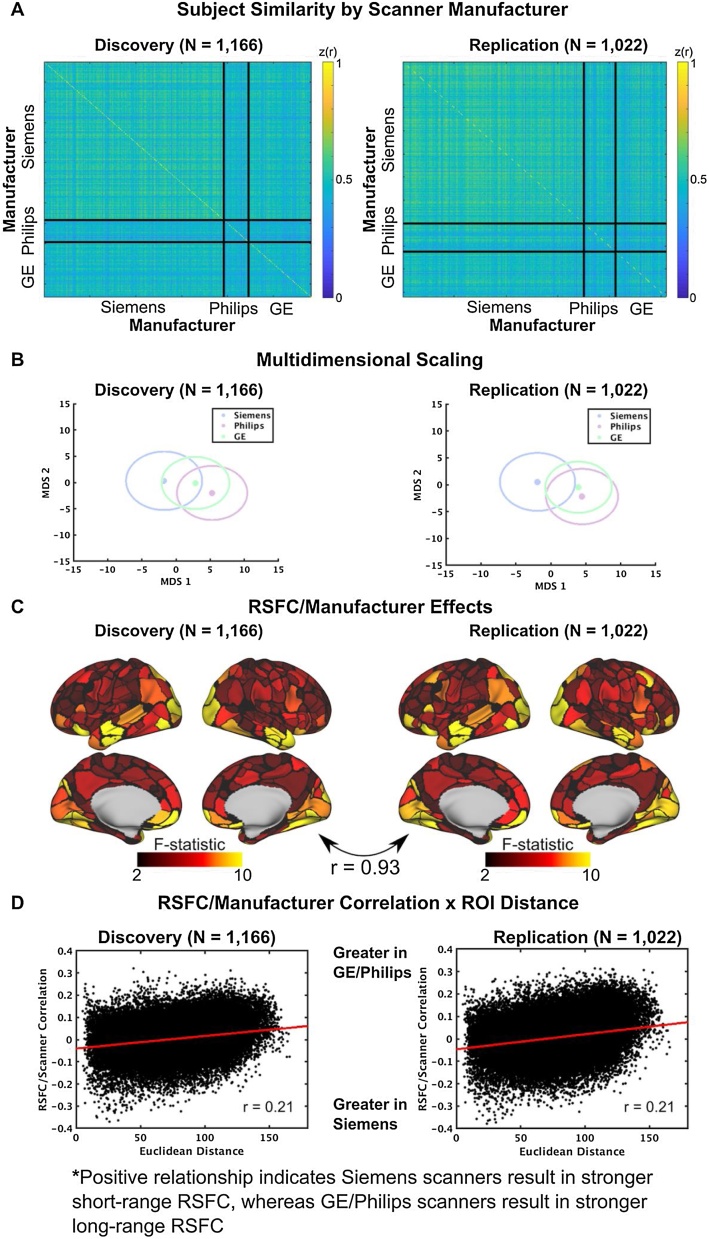
**Scanner manufacturer effects. (A)** RSFC similarity across individuals, sorted by scanner manufacturer (Siemens, Philips, GE). Each cell represents the whole brain correlation (similarity) between a pair of participants. Siemens scanners demonstrated higher similarity across participants than Philips or GE scanners. **(B)** MDS plots. Within these plots, each data point represents the mean across participants in multidimensional space, colored by the scanner manufacturer. Circles around the data points represent the 2-dimensional standard error of the mean. RSFC obtained with GE/Philips scanners are clearly dissociable from RSFC obtained with Siemens scanners **(C)** Correlations between RSFC and scanner manufacturer. Strong positive and negative correlations between the visual network and several other networks. **(D)** RSFC/scanner correlations demonstrate distance dependence, such that short-range ROI correlations, especially within the visual network, are weaker in GE/Philips scanners compared to Siemens, whereas long distance correlations are stronger in GE/Philips scanners compared to Siemens.

The ABCD dataset is uniquely suited to reveal population level variability in relationships between brain and behavior. We quantified potential variables that may covary with RSFC and behavior bias observed relationships between RSFC and general cognitive ability. We focused on three potential variables: data collection site, scanner manufacturer (e.g., Siemens, Philips, GE), and sex. For each variable, we used data-driven multivariate approaches to assess relationships between RSFC and these variables. Specifically, we employed similarity analyses (Gratton et al., 2018) and multidimensional scaling (see Material and Methods 2.8). These analyses revealed that across all participants and scanners, 49% of the variance in RSFC was shared across individuals, similar to the variance shared across adults (see Gratton et al., 2018). Similarity analyses revealed significant differences between scanner types (Fig. 2A), such that RSFC was more similar among participants acquired on Siemens scanners than those acquired on either Philips scanners (Δz(r) = 0.06, *p* < 0.001, *d* = 0.92) or GE scanners (Δz(r) = 0.05, *p* < 0.001, *d* = 0.73). Though significant differences were observed between participants scanned on Philips vs. GE scanners, the effect size was small in the discovery dataset (Δz(r) = 0.01, *p* <  0.001, *d* = 0.22) and negligible in the replication (*d* = 0.05). Jointly, these results suggest that there was a more general division between Siemens and Philips/GE.

Scanner-related effects were further quantified using multidimensional scaling (MDS). As can be observed in Fig. 2B, a significant portion of RSFC variance was attributable to scanner-related effects and was highly reproducible across the discovery and replication datasets. To probe these effects further, we tested scanner effects by conducting a one-way ANOVA of the three scanner types across individuals at each edge of the RSFC matrix and subsequently averaged all edges across ROIs, resulting in an average effect (F-statistic) of scanner manufacturer. In the discovery and replication dataset, there was a significant effect of scanner manufacturer in 24% of all edges (discovery: *F*_(2,1163)_ > 10.0*, p* < 0.001; replication: *F*_(2,1019)_ > 10.0*, p* < 0.001). At the ROI level, we observed a strong effect of scanner that was most notable in posterior brain regions, as well as in the anterior temporal lobe and orbitofrontal cortex (Fig. 2C).

Given the negligible to small differences between GE and Philips scanners, we collapsed these two manufacturers together and correlated RSFC (see below for comparison between each manufacturer), edge-wise, with manufacturer (Siemens vs. GE/Philips), enabling us to quantify the magnitude of the association for each ROI pair. Given previous spurious, distance-dependent developmental relationships between RSFC other confounding variables - namely head motion - we probed whether RSFC and manufacturer exhibited similar relationships with distance between ROI pairs. To this end, we correlated RSFC between each ROI pair with Euclidean distance between ROI centroids. The relationship between RSFC and manufacturer demonstrated a significant “short-to-long” effect, such that short-distance RSFC tended to be stronger in Siemens scanners relative to GE/Philips, whereas long-distance RSFC tended to be stronger in GE/Philips relative to Siemens scanners (*r* = 0.21, *p* <  0.001; results were equivalent in the discovery and replication dataset). Importantly, these scanner relationships with RSFC were on par with, or exceeded, those we found between RSFC and behavior (Fig. 5). Thus, we strongly suggest including manufacturer (Siemens, GE, Philips) as a nuisance variable in RSFC-behavior regression analyses (manufacturer main effects) and examining interactions between manufacturer and behavior.

Given that the observed “short-to-long” effect in scanner manufacturer effects resembled what has previously been reported for the effect of head motion (Power et al., 2012), we further examined the relative differences in subject head motion between manufacturers. We found a significant difference (p < 0.05) in average FD between subjects scanned in Siemens (Mean FD = 0.10) vs. Philips (Mean FD = 0.13) scanners and between Philips and GE (Mean FD = 0.10) scanners, but not between Siemens and GE scanners (*p* =  0.97). As such, we tested for scanner effects as a function of distance between each scanner manufacturer pair (Siemens vs. Philips, Siemens vs. GE, Philips vs. GE), while including mean FD as a covariate. Including mean FD as a covariate did not change the main conclusions drawn from Fig. 2D. Specifically, there was a significant distance-dependent effect for Siemens vs. Philips scanners, such that short-range RSFC was stronger for subjects scanned in Siemens scanners whereas long-range correlations were stronger for subjects scanned using Philips (discovery: *r* = 0.11, *p* <  0.001; replication: *r* = 0.13, *p* <  0.001). Similarly, there was a significant distance-dependent effect for Siemens vs. GE scanners, such that short-range RSFC was stronger for subjects scanned in Siemens scanners whereas long-range correlations were stronger for subjects scanned using GE (discovery: *r* = 0.23, *p* <  0.001; replication: *r* = 0.25, *p* <  0.001). There was a significant distance-dependent effect for Philips vs. GE scanners, such that short-range RSFC was stronger for subjects scanned in Philips scanners whereas long-range correlations were stronger for subjects scanned using GE (discovery: *r* = 0.06, *p* <  0.001; replication: *r* = 0.12, *p* <  0.001). Notably, while subjects acquired on Siemens and GE scanners exhibited no difference in head motion, the strongest RSFC/scanner distance dependent relationship existed between Siemens and GE scanners. As such, head motion alone cannot account for the distance-dependent RSFC/scanner relationships. We also tested whether altitude differences among sites may account for the observed scanner manufacturer effects. The observed effects of RSFC/scanner by anatomical distance associations were the same as those reported above when we (1) covaried for site altitude and (2) completely removed sites (n = 2) that were of substantially higher altitude than all others.

Given that head coils varied across manufacturers and sites (Table S1), we also tested for differences in head coils to examine whether these might account for the observed scanner manufacturer effects. In order to isolate potential head coil effects from the known manufacturer effects, we compared head coils in Siemens scanners only (32-channel vs. 64-channel). Similarity analysis revealed a small but significant difference between head coils, such that RSFC was more similar among participants acquired using 32-channel head coils vs. 64-channel head coils (discovery: Δz(r) = 0.02, *p* <  0.001, *d* = 0.26; replication: Δz(r) = 0.02, *p* <  0.001, *d* = 0.32). Though significant, the effect of head coil is substantially smaller than that of scanner manufacturer. Altogether, the results from these analyses suggest scanner manufacturer effects were relatively large, reproducible, and could not be easily explained by head motion, head coil, or site altitude.

**Fig. 3:**
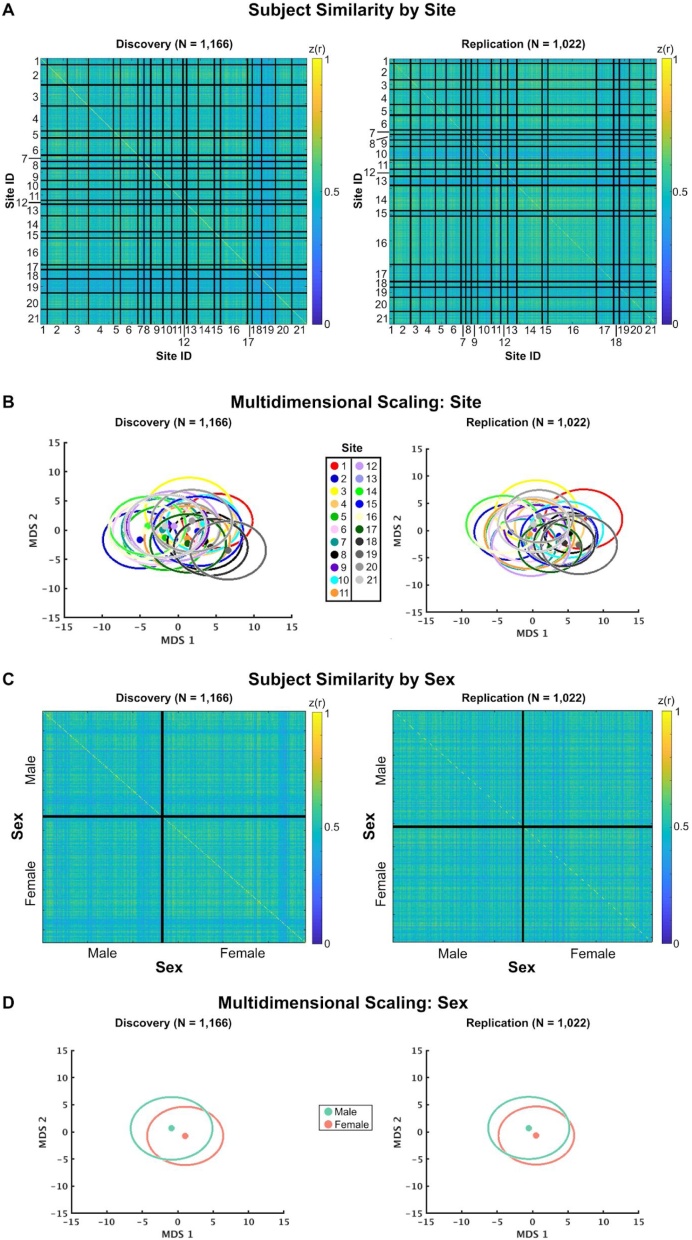
**Effects of scanning site and sex are small. (A & C)** RSFC similarity across individuals, sorted by scanning site (A) and sex (C). Note the strong homogeneity in similarity across scanning sites and sex, indicating a lack of evidence for whole brain site and sex effects. On average, sites 18 and 19 demonstrated the lowest similarity to other scanning sites. **(B & D)** Each data point represents the mean across participants in multidimensional space, colored by the scanner site in (B) and sex in (D). Circles around the data points represent the 2-dimensional standard error of the mean in multidimensional space. Scanner site and sex were not clearly captured within these dimensions, suggesting a lack of evidence for whole brain site effects and sex effects for ABCD resting-state data.

In contrast to scanner manufacturer, we observed much smaller effects on RSFC similarity when sorting by data collection site and sex (Fig. 3). We note however, some data collection sites are clearly separable in the MDS plots in Fig. 3B, such as differences between sites 1 & 2, 1 & 14, 2 & 19, and 14 & 19. Nevertheless, it is likely that some of these effects are driven by a combination of the scanner manufacturer effects (Fig. 2) and sample size differences across sites. RSFC was similar within (females: z(r) = 0.50; males z(r) = 0.49) and between (Δz(r) = 0.01, *d* = 0.27) sexes. The effect size of the difference between sexes was negligible in the replication set (Δz(r) = 0.005, *d* = 0.10), indicating RSFC globally is similar between males and females at this age. As the ABCD study continues, it will be critical to test whether the separation between males and females changes with age and pubertal status.

### 3.3 Between-participants variability in RSFC

**Fig. 4:**
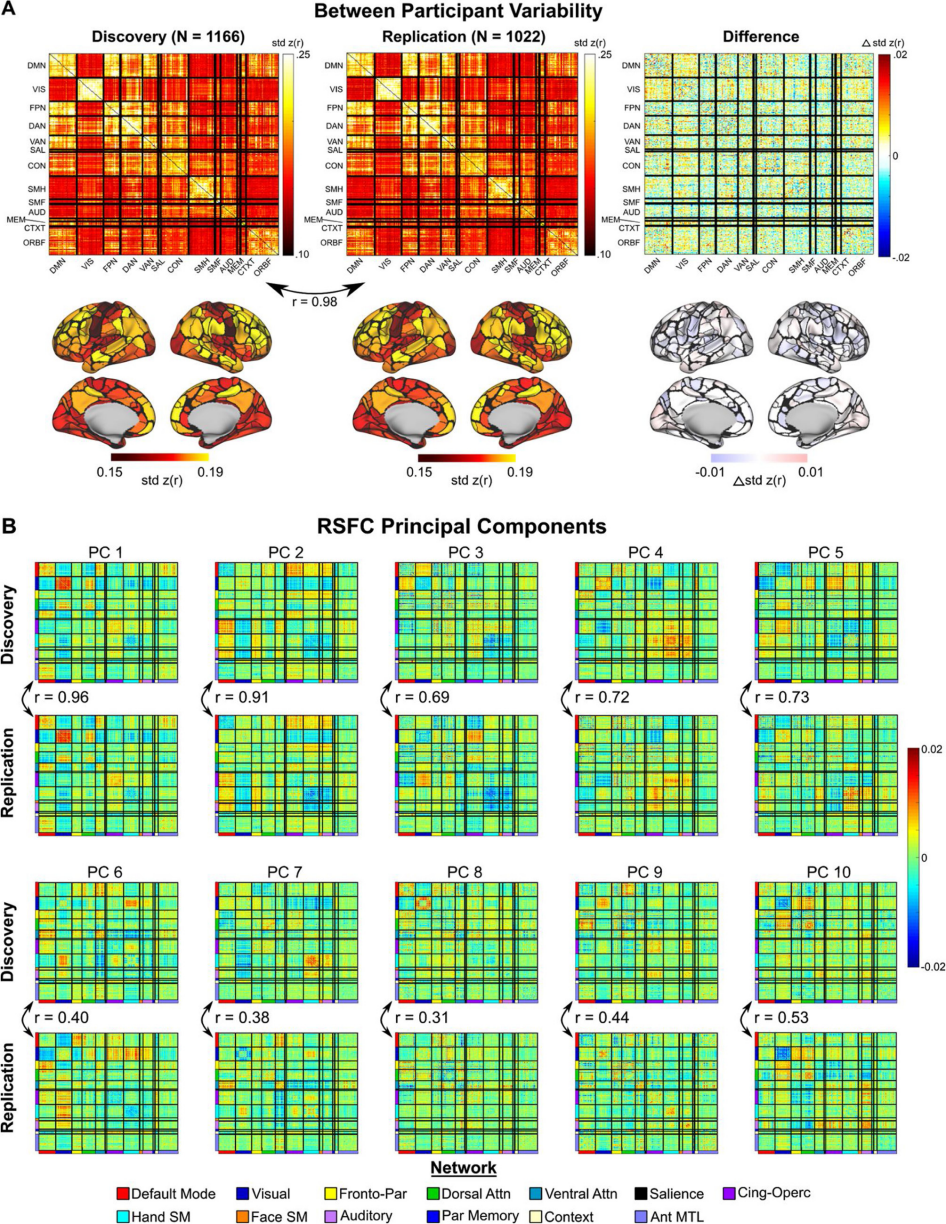
**Between-participant variance in RSFC. (A)***Top row:* Between-participants variability (standard deviation across participants) in RSFC for each ROI pair. Colorbar represents the magnitude of between-participants variability. *Bottom row*: Regional between-participants variability in RSFC, obtained by averaging the standard deviation of RSFC across all ROI pairs for each ROI. Similar to adults, children exhibit the greatest relative degree of between-participants variability within control and attention networks. **(B)** First 10 principal components of RSFC across participants in the discovery and replication datasets, accounting for 11.88% and 11.73% of the total variance across participants, respectively. Similar to prior work in adult samples (Smith et al., 2015), reproducible motifs of between participant variance are observed outside of traditional network architecture.

Individual differences in behavior are likely related to brain regions and networks that demonstrate a relatively larger degree of between-participant variability. Between-participant variability was generally larger for within-network connections, supporting a potential role of functional networks in individual differences in behavior. Beyond this observation and similar to adult studies, we found a high degree of between-participant RSFC variability in networks related to top-down control and attention (Fig. 4A), namely the frontoparietal and dorsal attention networks (*Msd* = 0.23 for both networks). Contrasting previous work in an adult sample (Gratton et al., 2018; Laumann et al., 2015), there was a large degree of between-participant variability within the visual network, that was on par with the frontoparietal and dorsal attention networks (*Msd* = 0.23). Although posterior brain regions that comprise the visual network exhibited large scanner effects, between-participant variance estimates were not driven by differences in scanner (all *d’s* < = 0.15 between scanner comparisons in both discovery and replication datasets; Fig. S3). Thus, within each scanner, between-participant variability within the visual network was similar to other control and attention networks and could not be explained by differences in scanner manufacturer. Rather, this variability may be driven by a general physiological property, such as high between-participant variability in arousal, as studies measuring eye movements indices of arousal have implicated regions of the visual network (Chang et al., 2016). Further supporting this idea, the first principal component of between-participant effects had high loadings on connections within the visual and default mode networks and those between default mode and dorsal attention networks (Fig. 4B). In addition to this component, other reproducible network motifs were observed in between-participant RSFC effects (see Fig. 4B for the first 10 principal components). Future studies can link dimensions of between-participant RSFC to other behavior measures, unrestricted from group-level network architecture.

**Fig. 5:**
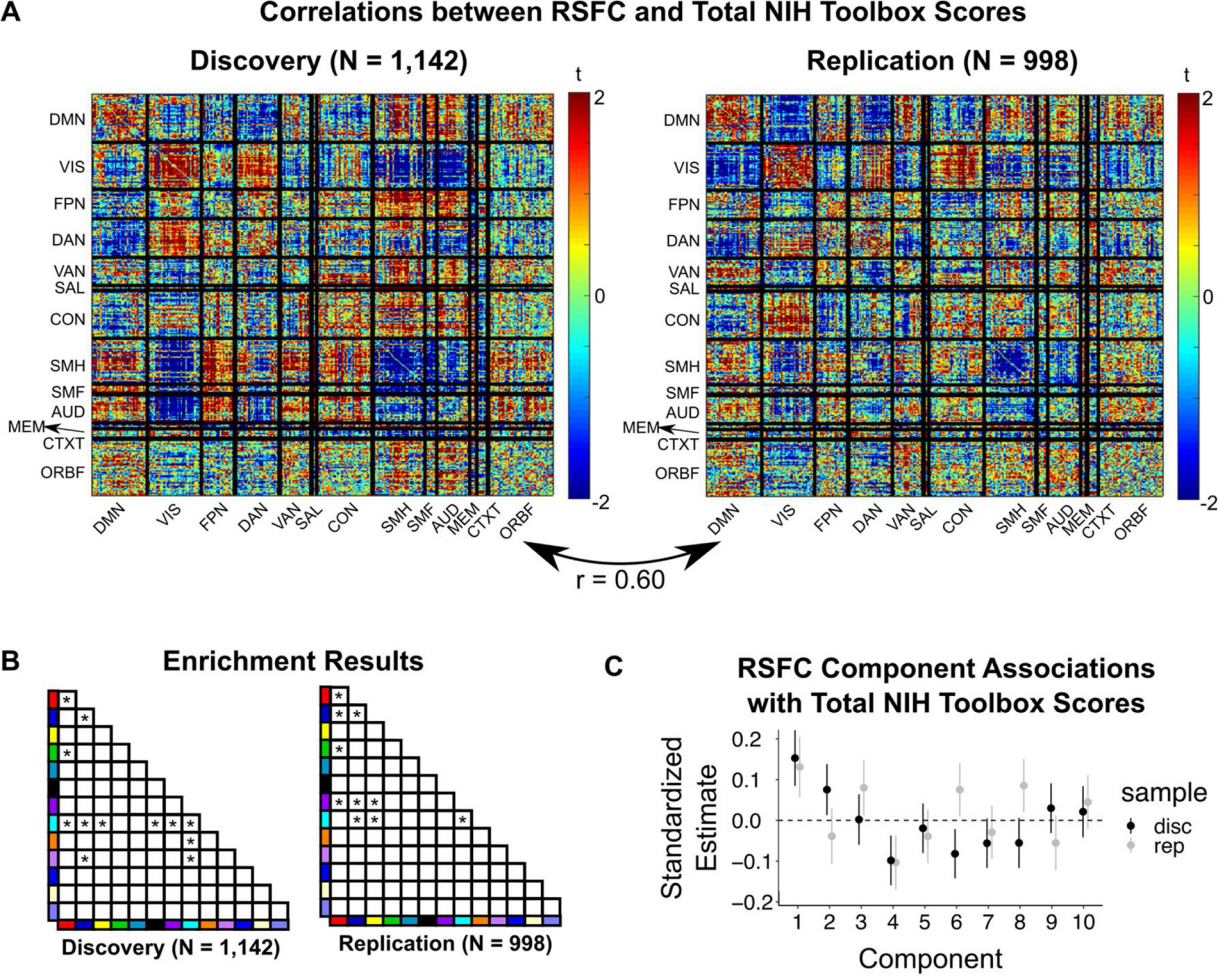
**RSFC-behavior correlations. (A)** Associations between RSFC and total scores on the NIH Toolbox. Edge-wise correlations exhibited split-half reliability of *r* = 0.60. **(B)** Enrichment analyses revealed several functional networks contribute to general cognitive ability. **(C)** Associations between RSFC principal components (reported in Fig. 4B) and total NIH Toolbox scores.

### 3.4 RSFC-behavior correlations

To demonstrate a framework through which brain-behavior correlations can be revealed and replicated, we correlated each ROI-ROI RSFC correlation with total scores on the NIH Toolbox within the discovery and replication datasets, separately. For each dataset, we determined network-level significance using enrichment analysis. Across all ROI pairs, the reliability (Pearson correlation) between the discovery and replication dataset was *r* = 0.60 (Fig. 5). Several network-level associations with general cognitive ability were revealed and replicated with the enrichment analysis, and were not driven by an interaction with scanner manufacturer, the largest source of potential bias identified in our initial analyses (mean R^2^ difference when including interaction term = 0.004; Fig. S4). Results were highly similar when including FD as a covariate (correlation between model including FD and model not including FD: discovery: *r* = 0.98; replication *r* = 0.99). Significant associations were exhibited between several network pairs, including the association between DMN-DAN RSFC and general cognitive ability (discovery: *p* = 0.01; replication: *p* < 0.001), such that greater anticorrelation between the DMN and DAN was associated with higher general cognitive ability. Additionally, stronger between-network FPN-SMH RSFC was associated with higher general cognitive ability (discovery: *p* < 0.001; replication: *p* = 0.009). Within networks, we found that stronger RSFC within the DMN (discovery: *p* = 0.008; replication: *p* < 0.001) and visual networks (discovery: *p* < 0.001; replication: *p* < 0.001) also was associated with greater cognitive ability. Supporting these observations, we found that the first principal component of between-participant effects (Fig. 4B), which had high positive loadings on connections within DMN and visual networks and broadly distributed loadings on between-network connections, was significantly associated with greater cognitive ability in both discovery (p < 0.001) and replication sets (p < 0.001). Jointly, these results indicate that widely distributed circuitry within and between several functional networks is associated with general cognitive ability in childhood.

## 4 Discussion

Using initial data from the developmental ABCD dataset, this study quantified functional brain networks in children and examined network associations with individual differences in general cognitive ability, while also examining potential confounding effects within the multi-site, multi-scanner project. Consistent with prior work (Grayson et al., 2014; Marek et al., 2015; Power et al., 2012), our results support the presence of adult-like functional networks in children, which were highly reproducible across discovery and replication samples. Less consistency was observed across scanner manufacturers (i.e., Siemens, GE, Philips), where a distance-dependent relationship was observed between RSFC and scanner manufacturer. When accounting for these confounding effects, our results suggest widely-distributed circuitry is associated with general cognitive ability, including previously highlighted DMN-DAN anticorrelations (Shine et al., 2019; Smith et al., 2015). Taken together, these results provide a critical resource and framework for ongoing work with the ABCD dataset and efforts to link functional networks to trait-level behavioral features.

### 4.1 RSFC and functional network architecture are highly reproducible

Across datasets, group-level RSFC was highly reproducible (r > 0.99), and generally consistent across males and females, as well as scanning sites. Methodologically, this observation strongly supports the multi-site harmonization efforts in the ABCD project and other multi-site neuroimaging projects. We observed that functional network architecture was highly reproducible (NMI = 0.98) and shares many features of adult-level networks. We did note differences between the group-average ABCD parcellation and the adult level parcellation from Gordon et al., 2017; however, the vast majority of these differences were along network borders, likely resulting from differences in alignment procedures rather than neurobiology. As such, paralleling findings by Marek et al., 2015, the adult-level spatial arrangement in cortical functional networks (i.e., functional network organization) is present prior to the onset of adolescence.

The excellent reproducibility of RSFC within this age group will make for a reliable resource through which to compare and contrast future years of ABCD data collection. In this way, developmental neuroscientists will be able to delineate even small magnitude effects in population level brain development throughout adolescence. Previous studies have detailed robust maturation of within- and between-network correlations (Gu et al., 2015; Marek et al., 2015), as well as widely distributed RSFC (Nielsen et al., 2018). For example, several studies have noted functional network maturation disproportionately implicates the cingulo-opercular control network and somatomotor networks, which in turn supports the maturation of inhibitory control (Fair et al., 2012; Grayson et al., 2014; Marek et al., 2015). Future years of the ABCD study will be able to precisely characterize maturational RSFC profiles in a longitudinal fashion, providing a powerful resource for normative adolescent growth curves of RSFC.

### 4.2 Scanner manufacturer effects

In contrast to the relatively minimal effects of sex and acquisition site, we observed robust and systematic differences corresponding to scanner manufacturer. Specifically, there was a distance-dependent relationship between RSFC and manufacturer, such that short-range ROI correlations, especially within the visual network, are weaker in GE/Philips scanners compared to Siemens, whereas long distance correlations are stronger in GE/Philips scanners compared to Siemens scanners. However, we note that although greater similarity exists within subjects scanned on Siemens scanners compared to those scanned on Philips and GE scanners, it is possible that systematic biases across sites using Siemens scanners may still exist. As such, we encourage investigators to probe other factors that may contribute to systematic biases within each scanner type, including the analysis of a traveling subject and/or phantoms. Adequately addressing these potentially confounding effects of scanner manufacturer is essential for moving developmental cognitive neuroscience toward reproducible findings, and more systematic research into the potential source of these effects is warranted. Given these results, we strongly advocate that researchers using the ABCD dataset make efforts to control for manufacturer effects. Here, we employed one possible approach, by testing for interactions between a behavioral variable of interest and scanner manufacturer, in order to ensure that reported effects were not driven by differences across manufacturers. Recent machine learning approaches, such as ComBat (Fortin et al., 2018, 2017; Johnson et al., 2007; Yu et al., 2018), may be powerful alternatives to controlling for batch effects. In genomics, use of ComBat has been shown to remove manufacturer effects. ComBat has also been shown to remove MRI manufacturer effects better than other approaches. Future work should also investigate MRI signal properties that contribute to the observed manufacturer effects in more detail.

### 4.3 Sources of individual variability reside within and outside conventional network architecture

The highest inter-individual differences were observed within networks, where resting-state correlations tend to be the highest. Beyond this observation, consistent with studies in adults (Finn et al., 2015; Gratton et al., 2018; Laumann et al., 2015; Mueller et al., 2013), the highest levels of individual variability were observed in control networks (Dosenbach et al., 2008, 2006; Marek and Dosenbach, 2018), such as the frontoparietal and dorsal attention networks, as well as the default mode network. Other between-network associations, such as between the DMN and frontoparietal networks and between the DMN and DAN also exhibited relatively larger inter-individual variability. The observation of a high degree of inter-individual variability within and between these networks raises the possibility that trait-level behavioral features are associated with broad differences in RSFC that may or may not respect functional network boundaries. Supporting this, principal component analysis performed on individual differences in RSFC correlation matrices suggests the largest sources of variation were typically explained by commonalities across multiple-networks. This observation warrants future consideration in both theory and empirical research, as the current project adds to a growing literature highlighting robust between-participant differences that span multiple networks (Smith et al., 2015; Xia et al., 2018). Future studies should determine developmental change in this pattern of variability. In addition to higher-order networks, we observed high inter-individual variability within the visual network, which we hypothesize may be related to a broad physiological property like arousal (Chang et al., 2016). Both the amplitude of the BOLD signal and correlations between regions are sensitive to changes in arousal state (Larson-Prior et al., 2009; Tagliazucchi and Laufs, 2014), and previous studies have found increases in BOLD signal fluctuation in the visual cortex (Esposito et al., 2014; Horovitz et al., 2008) and between the DMN and DAN (Chang et al., 2013; Esposito et al., 2014). However, further research into the underlying mechanisms of patterns of between-participant variability in RSFC is warranted.

### 4.4 RSFC-behavior associations

Conceptually, our results support the notion of a primary role of both within- and between-network interactions relating to individual differences in cognition (Anticevic et al., 2012; Gao et al., 2013; Sherman et al., 2014; Shine et al., 2019; Smith et al., 2015). Developmental studies have found that segregation of the DMN from other control and attention networks increases throughout development and is related to general cognition throughout development (Sherman et al., 2014). Our results from both network and PCA approaches highlighted a more widely-distributed circuitry of control, attention, and motor networks, including RSFC between the frontoparietal and somatomotor networks, as well as within visual network connections. However, one must interpret these findings with care as many demographic and cognitive variables are associated with head motion (Siegel et al., 2017). As such, the current study employed rigorous head motion correction procedures in an attempt to address the potential confounds of head motion. Altogether, these findings support a broader cognitive literature that has begun to link widely-distributed patterns of RSFC to cognition. These results are critical for the aims of the ABCD study as individual differences within control regions have been associated with precocious substance use (Heitzeg et al., 2015; Tervo-Clemmens et al., 2018) and across multiple forms of psychopathology (McTeague et al., 2017).

### 4.5 Limitations

This project is characterized by a number of strengths, including a large sample size, characterization of potential confounding variables that are inherent to the ABCD project and other multi-site study designs, and demographically-matched split-halves for replication. However, it is worth noting some limitations. First, although we explored reproducibility via split-halves of the data, we did not use more complex k-fold cross-validation techniques, which may result in higher reproducibility. In any case, split-half analyses were needed to precisely estimate demographically-match split-halves within the dataset. Future work may investigate more fine grained analysis of reproducibility and its variability (Lusseau et al., 2008). Second, our brain-behavior analyses focused on only one, albeit highly hypothesized, individual difference variable (general cognitive ability). Therefore, future work may examine the reproducible brain-behavior patterns form the current project in the larger context of cognitive variables. Third, this study focused on RSFC data; similar studies should focus on task-based fMRI and structural data. Finally, the current study did not include subcortical regions or the cerebellum, which also support cognitive function and undergo developmental changes in RSFC (Greene et al., 2014; Guell et al., 2018a, 2018b; Marek et al., 2018; Schmahmann, 2019).

## 5 Conclusion

The ABCD dataset provides researchers the opportunity to characterize population-level development of neural features and relate those features to behavioral development. The current work characterized RSFC and network architecture in the child brain, quantified several key potential confounding variables inherent to large-scale multi-site study designs, and provided a framework for producing rigorous and reproducible brain-behavior associations. We found RSFC and network architecture was highly reproducible across children and conformed to many features observed in adult-level networks. Supporting the harmonization efforts across ABCD sites, we did not observe strong site effects. However, scanner manufacturer effects were large and reproducible; as such, we suggest accounting for scanner effects in future analyses of ABCD data. Accounting for potential confounding variables, we revealed and replicated that RSFC between several higher-order networks was related to general cognition. Altogether, this work provides a framework for characterizing brain function and its relationship to behavior in a rigorous and reproducible manner using the ABCD dataset.

## Funding

This work was supported by the National Institutes of Mental Health, including T32 MH100019 (SM MDW), R01MH067924 (BL), R03MH113090 (BTC), K01MH103594 (ATE), U01DA041120 (DMB), NS088590 (NUFD), TR000448 (NUFD), R01 MH115357 (DAF), 1R25MH112473 (TOL), R01MH115357-02S1 (RH), K01MH104592 (DJG), U01 DA041148 (BJN, DAF, SWFE), U24 DA04112 (AMD), R01 MH096773 (DAF), R01 MH115357 (DAF), R01MH105538 (DAF), U01 DA041148 (BJN, DAF, SWFE), U24 DA04112 (AMD), 1P30NS098577 (to the Neuroimaging Informatics and Analysis Center), and HD087011 (to the Intellectual and Developmental Disabilities Research Center at Washington University); the Jacobs Foundation grant 2016121703 (NUFD); the Child Neurology Foundation (NUFD); the McDonnell Center for Systems Neuroscience (NUFD); the Mallinckrodt Institute of Radiology grant 14-011 (NUFD); the Hope Center for Neurological Disorders (NUFD); National Library of MedicineT15LM007088 (EF).
