# Supplementary material for: Identifying reproducible individual differences in childhood functional brain networks: An ABCD study
Source: Dev Cogn Neurosci. 2019 Sep 19;40:100706. doi: 10.1016/j.dcn.2019.100706 (PMC6927479; doi:10.1016/j.dcn.2019.100706)
Supplement: Supplementary file 1 [file mmc1.docx]

**SUPPLEMENTAL TABLES**

**Table S1. Scanner Details**

| **Site** | **Scanner** | **Head Coil (channel #)** |
| --- | --- | --- |
| 1 | Philips Achieva dStream | 32 |
| 2 | SIEMENS Prisma fit | 32 |
| 3 | SIEMENS Prisma | 32 |
| 4 | GE DISCOVERY MR750 | 32 |
| 5 | SIEMENS Prisma fit | 32 |
| 6 | SIEMENS Prisma fit | 32 |
| 7 | SIEMENS Prisma fit | 64 |
| 8 | GE DISCOVERY MR750 | 32 |
| 9 | SIEMENS Prisma fit | 32 |
| 10 | GE DISCOVERY MR750 | 32 |
| 11 | SIEMENS Prisma | 64 |
| 12 | SIEMENS Prisma fit | 64 |
| 13 | GE DISCOVERY MR750 | 32 |
| 14 | SIEMENS Prisma fit | 32 |
| 15 | SIEMENS Prisma fit | 64 |
| 16 | SIEMENS Prisma  SIEMENS Prisma fit | 32 |
| 17 | Philips Achieva dStream | 32 |
| 18 | GE DISCOVERY MR750 | 32 |
| 19 | Philips Ingenia | 32 |
| 20 | SIEMENS Prisma  SIEMENS Prisma fit | 32 |
| 21 | SIEMENS Prisma  SIEMENS Prisma fit | 32 |

**SUPPLEMENTAL FIGURES**

**
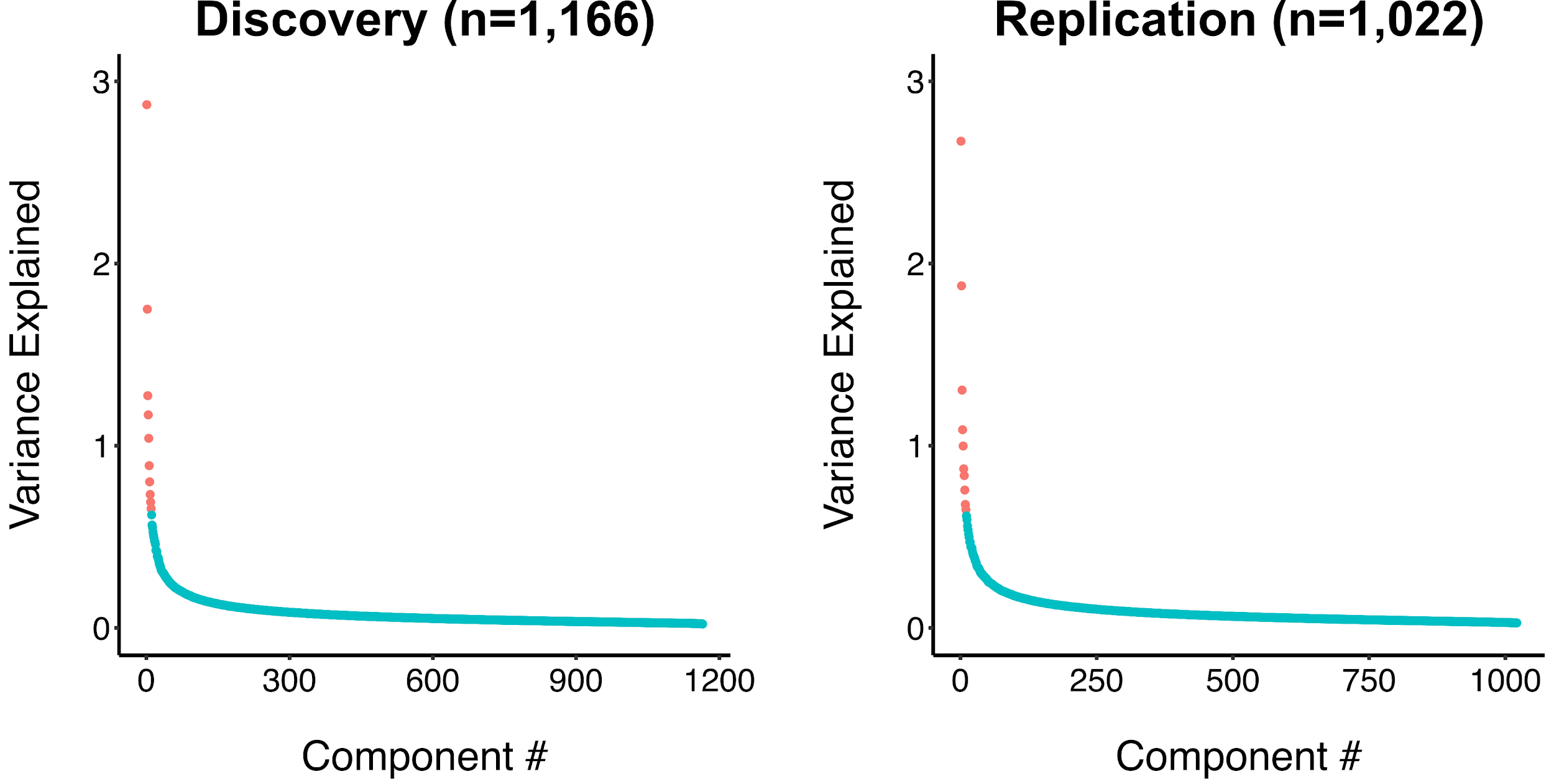
**

**Figure S1. Variance Explained in PCA of Between-Subject RSFC Data.** Red dots indicate the first ten components, which we present in the current project and explained 11.88% and 11.73% of the total variance in discovery (left) and replication (right) data sets, respectively.


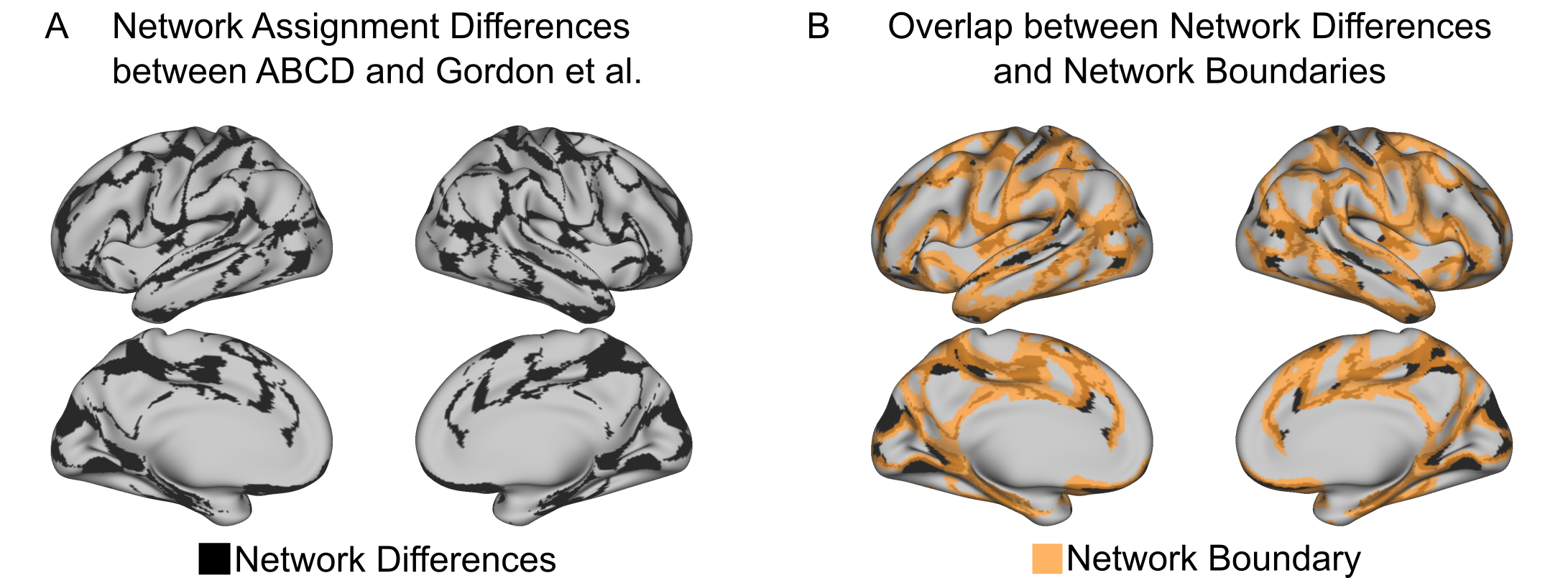


**Figure S2. Network assignment comparison between ABCD and Gordon *et al*., 2017. (A)** Vertex assignments that differ between ABCD and Gordon *et al.* (black). **(B)** Network boundaries from Gordon et al (orange) overlaid on vertices that differed in network assignment between ABCD and Gordon et al (A). Note the high overlap between network boundaries and network differences.

**
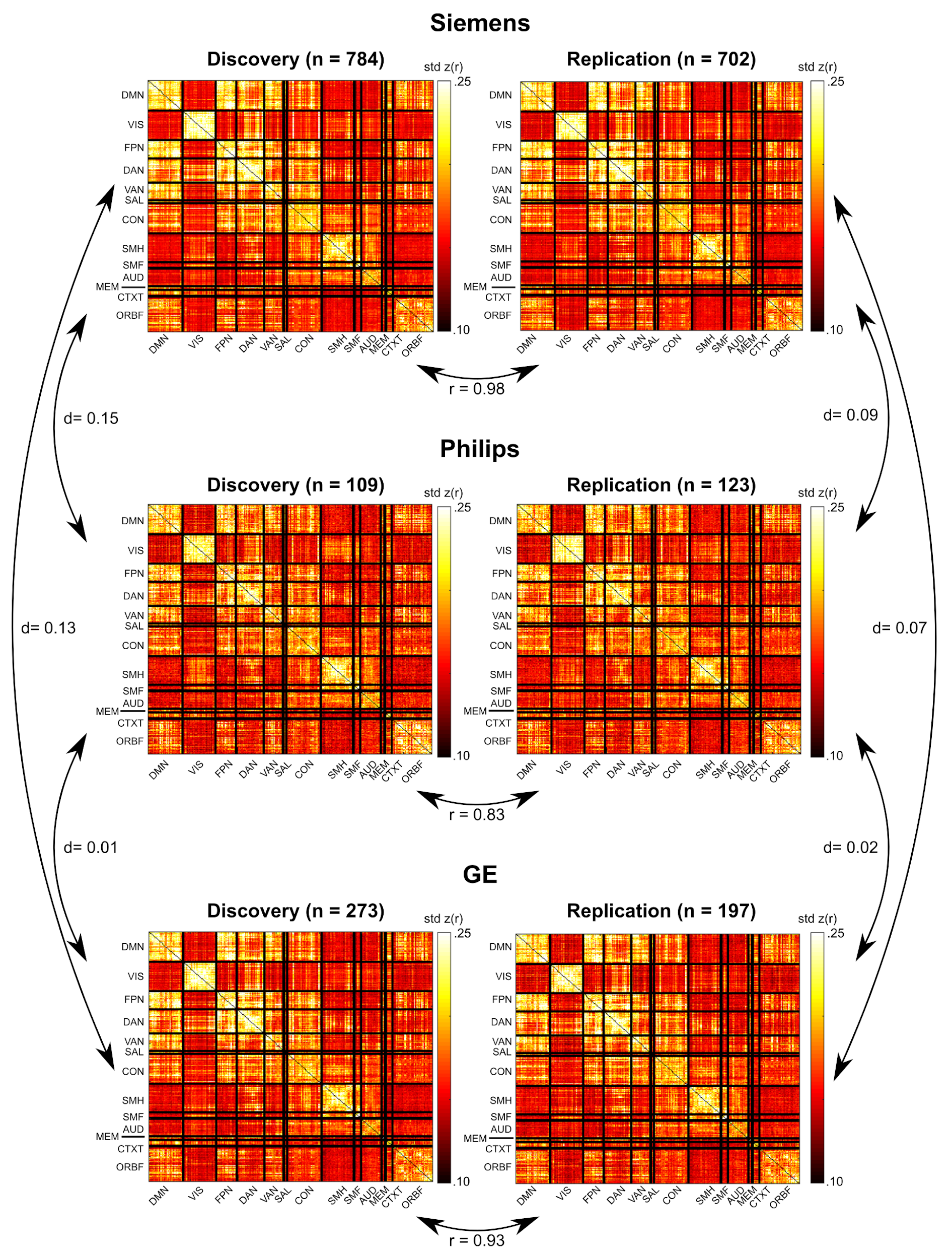
**

**Figure S3. Between-participant RSFC variability is not driven by scanner manufacturer.** Between-participant RSFC variability for Siemens, Philips, and GE scanners separately. There was a negligible effect of scanner on between-participant RSFC variability (all pairwise comparisons: *d* <= 0.15). RSFC variability also was highly reproducible within scanner (lowest *r* = 0.83, observed across Philips scanners).


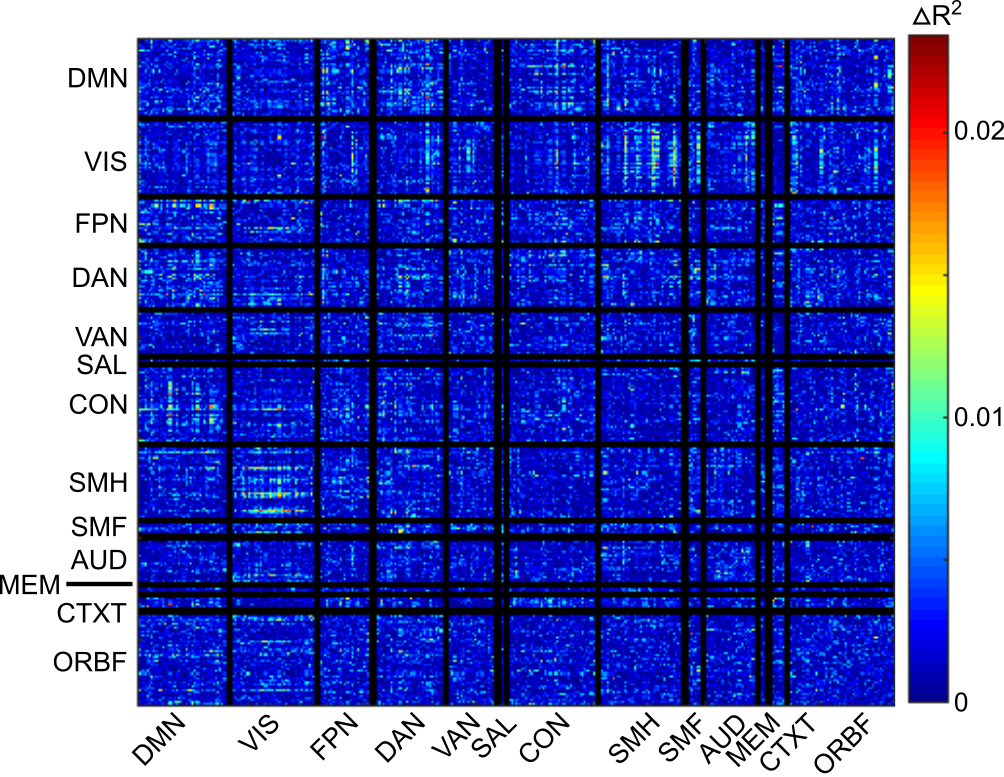


**Figure S4. RSFC-cognition effects were not driven by an interaction with scanner manufacturer.** Each pairwise RSFC-cognition association was tested for potential additive effects of an interaction between scanner manufacturer and cognition. Each entry in the matrix is the difference in R^2^ between a model including a main effect of scanner manufacturer and a model including the interaction between cognition and scanner manufacturer.
